# The Myosuit: Bi-articular Anti-gravity Exosuit That Reduces Hip Extensor Activity in Sitting Transfers

**DOI:** 10.3389/fnbot.2017.00057

**Published:** 2017-10-27

**Authors:** Kai Schmidt, Jaime E. Duarte, Martin Grimmer, Alejandro Sancho-Puchades, Haiqi Wei, Chris S. Easthope, Robert Riener

**Affiliations:** ^1^Sensory-Motor Systems Lab, Department of Health Sciences and Technology, Institute of Robotics and Intelligent Systems, ETH Zurich, Zurich, Switzerland; ^2^Spinal Cord Injury Center, University Hospital Balgrist, University of Zurich, Zurich, Switzerland

**Keywords:** Myosuit, exomuscle, exosuit, anti-gravity, assistance, wearable, textile, muscle-activity

## Abstract

Muscle weakness—which can result from neurological injuries, genetic disorders, or typical aging—can affect a person's mobility and quality of life. For many people with muscle weakness, assistive devices provide the means to regain mobility and independence. These devices range from well-established technology, such as wheelchairs, to newer technologies, such as exoskeletons and exosuits. For assistive devices to be used in everyday life, they must provide assistance across activities of daily living (ADLs) in an unobtrusive manner. This article introduces the Myosuit, a soft, wearable device designed to provide continuous assistance at the hip and knee joint when working with and against gravity in ADLs. This robotic device combines active and passive elements with a closed-loop force controller designed to behave like an external muscle (exomuscle) and deliver gravity compensation to the user. At 4.1 kg (4.6 kg with batteries), the Myosuit is one of the lightest untethered devices capable of delivering gravity support to the user's knee and hip joints. This article presents the design and control principles of the Myosuit. It describes the textile interface, tendon actuators, and a bi-articular, synergy-based approach for continuous assistance. The assistive controller, based on bi-articular force assistance, was tested with a single subject who performed sitting transfers, one of the most gravity-intensive ADLs. The results show that the control concept can successfully identify changes in the posture and assist hip and knee extension with up to 26% of the natural knee moment and up to 35% of the knee power. We conclude that the Myosuit's novel approach to assistance using a bi-articular architecture, in combination with the posture-based force controller, can effectively assist its users in gravity-intensive ADLs, such as sitting transfers.

## 1. Introduction

Mobility and independence are key determinants for quality of life (Schalock, 2004; Pukeliene and Starkauskiene, 2011). When mobility is limited, a person's quality of life can be impacted negatively. The main causes for mobility limitations are reductions in physical capacity with increasing age (Kalache and Kickbusch, 1997), diseases, or injuries. From the age of 60 to the age of 85, the mean steps per day decrease by about 77% (Tudor-Locke et al., 2013). This can negatively impact a person since a higher number of steps per day is associated with several positive health outcomes including reductions in body mass index (Bravata et al., 2007), risk of cardiovascular disease (Murtagh et al., 2010), and mortality (Erlichman et al., 2002). Along with aging, neurological injuries such as spinal cord injury (SCI) can also limit a person's mobility. For example, incomplete SCI patients (ASIA grades C and D; 30% of SCI patients) have full range of motion and the ability to move against gravity with at least half of the key muscles. However, walking (64%) and standing (25%) abilities, which are top priorities for this patient group (Brown-Triolo et al., 2002), are clearly limited for most of the patients (Barbeau et al., 1999).

While exercise can help mitigate the reductions in strength (Gross et al., 1998) and stamina (Talbot et al., 2000) with increasing age, the overall trend cannot be stopped (Ades et al., 1996; Talbot et al., 2000). The best option for many people to be mobile is then the use of assistive technologies. These technologies range from passive devices, such as orthoses or wheelchairs, to powered devices, such as exoskeletons. In contrast to passive devices, powered systems can compensate for the functional loss of strength and stamina. Devices such as the Re-Walk (Esquenazi et al., 2012) or the Indego (Quintero et al., 2011) use rigid structures, in parallel to the user's legs, and electric motors to stabilize the human against gravity during standing and walking. Thus far, these systems have been used mostly in clinical environments for gait rehabilitation (Federici et al., 2015). Their weight, which can range from 13 to 48 kg (Quintero et al., 2011; Kilicarslan et al., 2013), can make them difficult to use and transport, thus limiting their applicability beyond clinical environments.

A new breed of devices, which move away from rigid structures, are exosuits. These devices use textile structures to interface with the human body and can be made significantly lighter and more portable. Exosuits were initially designed to reduce user effort during walking while carrying heavy loads (Asbeck et al., 2013). A system that supports the hip (i.e., flexors) and ankle joint (i.e., planatar flexors) was developed to facilitate ground-level walking and weighs about 5.5 kg (Asbeck et al., 2014). A similar system that provides additional support of the hip extensors (6.5 kg in weight) is able to assist with forces corresponding to torques of 21 and 19% of the nominal biological joint torques at the ankle and hip during unloaded walking (Asbeck et al., 2015). About 23% of metabolic reductions have been achieved with such a multi-articular suit architecture (hip flexion and ankle plantar flexion) for non-loaded level ground walking using a tethered system (Quinlivan et al., 2017). An exosuit that assists the ankle movement of post-stroke patients has been developed for mobility assistance (Bae et al., 2015; Awad et al., 2017). This exosuit supports the ankle joint in plantar and dorsi flexion in one leg and weighs about 4.1 kg in total. The system was shown to reduce the metabolic burden associated with post-stroke walking by 32% using only relatively low assistance of about 12% of the biological joint torques (Awad et al., 2017). Yet another exosuit design that assists the swing phase during walking of elderly subjects has shown to reduce energy expenditure by 5.9% by applying small forces at the hip joint (24.5 N, corresponding to ~8% of biological joint torque assuming a 20 cm moment arm) (Jin et al., 2017). While these devices have demonstrated the ability of exosuits to assist in the forward propulsion of walking, to our knowledge, there is no exosuit that can assist the user in supporting his weight against gravity. They also have limited capabilities when supporting other activities of daily living (ADLs) such as stair climbing and sitting transfers where anti-gravity muscles play a dominant role (Winter, 1984; Anderson and Pandy, 2003).

This article introduces a new exosuit design concept: the Myosuit. The Myosuit is a wearable robotic device designed to address the functional aim of most exoskeletons–provide anti-gravity support during standing, walking, and sitting transfers–with the soft textile interface featured in exosuits. Its design is based on the analysis of kinematic and kinetic data, along with muscle activation patterns, to identify the synergy sequences involved in supporting the body against gravity during ADLs (Bartenbach et al., 2015; Schmidt and Riener, 2017). Based on this analysis, and in accordance with previous literature, the knee and hip extensor muscles were identified as the primary anti-gravity muscles of the leg. A previous study has already shown that muscle activity can be reduced in these muscles (He and Kiguchi, 2007). In this study, assistance was provided during gravity-intense movements such as sitting transfers with a rigid exoskeleton using mono-articular actuators.

Exosuits can be designed in such a way that their actuators encompass multiple joints, mimicking bi-articular muscles which apply forces to adjacent joints at the same time (for a thorough review on bi-articular elements see Junius et al., 2017). This means that an exosuit can have a simpler mechanical design by applying forces to two joints using only one motor. Asbeck et al. used a multi-articular strategy to assist hip flexion and ankle planar flexion during ground-level walking (Asbeck et al., 2013). The Myosuit uses yet another bi-articular approach that supports hip and knee extension. In these bi-articular configurations the magnitude of the transmitted torques is strongly dependent on the size of the moment arm across the targeted joint. This feature allows for the regulation of the transmitted torques while applying the same muscle force (Winter, 2009). Based on this concept, and a novel posture-based control approach, the Myosuit delivers continuous assistance to the user when moving against gravity (e.g., getting up from a chair), when moving with gravity (e.g., sitting on a chair) or when holding an upright posture (e.g., standing). We hypothesize that the Myosuit can reduce the muscle activity of knee and hip extensor muscles—specifically gluteus maximus and vastus lateralis—during sitting transfers by providing bi-articular force assistance solely based on the knee angle. We conducted an initial study with a single subject performing sitting transfers and showed that the muscle activity of hip extensors was reduced when the assistive forces were applied. Therefore, we conclude that the Myosuit's novel approach to assistance: a bi-articular architecture combined with continuous force control, can effectively assist its users in gravity-intensive ADLs.

## 2. Materials and methods

### 2.1. The myosuit concept

**Figure 1:**
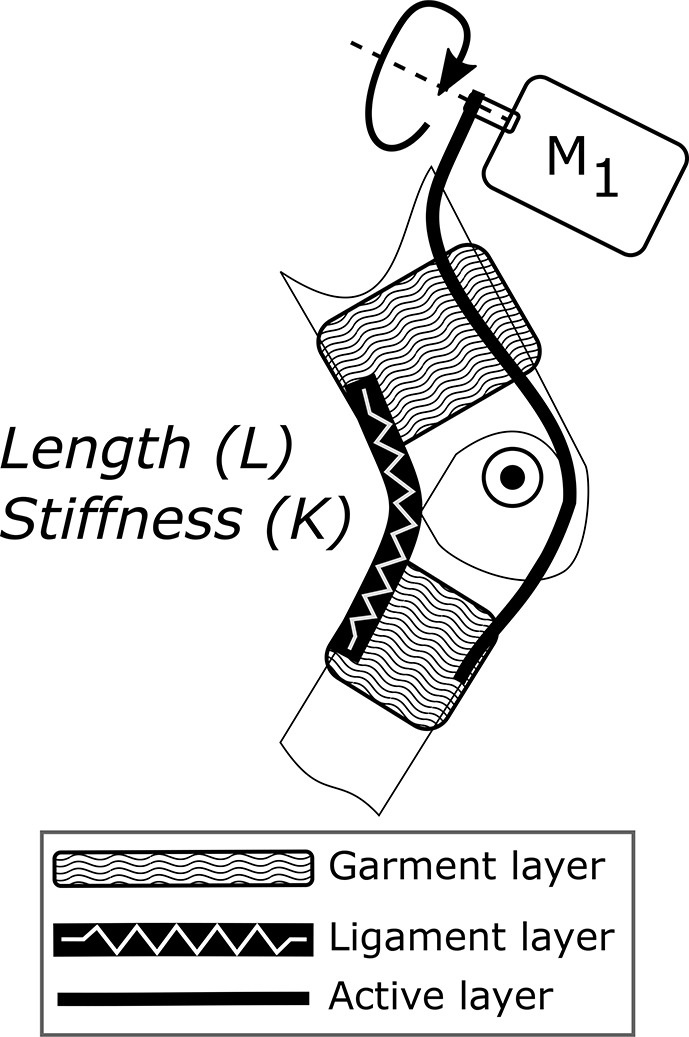
Three-layer architecture of the Myosuit. The architecture is inspired by the bones (structural support), ligaments (passive support), and muscles (active support) of the human's musculoskeletal system. The garment layer is the interface between the Myosuit and the user and provides the overall structure of the suit. The ligament layer incorporates passive elements—rubber bands—to store energy and passively assist the joint's movement. The power layer uses an actuator, routed along the limb, to actively assist the movement of the joint.

**Figure 2:**
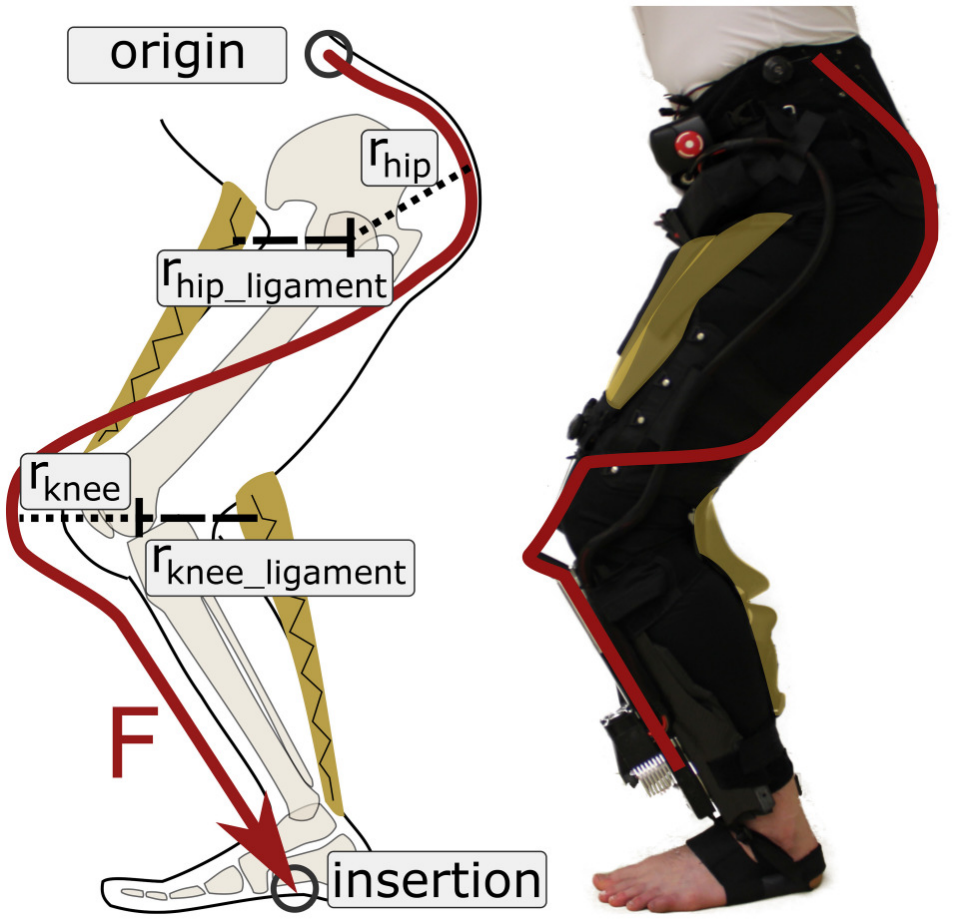
Active and ligament layers of the Myosuit. The Myosuit's tendon actuators are attached at the shank and anchored to a waist belt at the hip and by a wrap at each foot. The tendons are routed along the leg using textile cable channels sewn to the garment layer. When the support forces are active, the tensile forces translate into extension torques at the hip and knee joint. The ligament layer provides an antagonistic structure to assist with hip and knee flexion. In this study, which focused on sitting transfers, the ligament was attached to the Myosuit, but not tensioned.

The Myosuit is a textile-based robotic device designed to assist people with mobility impairments when performing activities of daily living (ADLs). Most exosuits transmit forces—generated by actuators—to a user through the interaction of garment-like, functional textile anchors and cable-based transmissions (Asbeck et al., 2015; Awad et al., 2017; Jin et al., 2017). The Myosuit extends this concept by classifying a three-layer architecture, which is inspired by the bones, ligaments, and muscles of the human's musculoskeletal system, to dynamically adapt the levels of support according to the user's needs. The three layers are: a garment layer, a ligament layer, and a power layer (Figure 1). The garment layer is the interface between the Myosuit and the user and provides the overall structure of the suit and distributes the forces along the body. The ligament layer incorporates passive elements to store energy and passively assist with hip and knee flexion. The power layer uses one actuator and an artificial tendon, routed along the leg from the shank to the waist, to actively assist against the force of gravity at the hip and knee joint (Figure 2). The three layers of the Myosuit have been designed to work similar to an antagonistic pair of muscles to modulate the forces and the stiffness around the biological joints, and thus, provide structural stability in the absence of a rigid frame.

The Myosuit provides assistance that goes beyond event-triggered bursts of forces relying on specific phases of the gait cycle. Instead, it provides forces continuously either with gravity (damping—eccentric behavior) or against gravity (concentric behavior) throughout the user's movements. The forces are based on the joints' posture, thus resembling the behavior of biological muscles. When the forces in the power layer are scaled down due to decreasing influence of gravity, the transmission of mechanical work of the passive elements in the ligament layer provide support during hip and knee flexion. This force-based approach is made possible by running an embedded, real-time system at 1 kHz that uses closed-loop force control instead of a position based control approach.

The Myosuit combines the assistance aim of most lower limb exoskeletons—anti-gravity support—with the lightweight design of the textile interfaces of exosuits. The total mass of the current system is 4.09 kg without batteries and 4.56 kg including lithium polymer batteries that can power the system up to 4 h. The assistance is partially based on a kinematic and kinetic analysis of limb synergies involved in ADLs (Bartenbach et al., 2015). Limb synergies are defined as phases during a movement where the joints collaborate toward a common goal. These synergies were examined regarding their consistency in either power generation or absorption assuming that the power of coupled joints must be either positive or negative at the same time. This is based on the property of tensile actuators that are only able to contract (generate force) or extend (damping) concurrently. Two main synergies were identified for the lower limbs during ground-level walking: a synergy that supports forward propulsion, and a synergy that acts against the force of gravity. The first synergy, which focuses on hip flexion and ankle plantarflexion, has been successfully used in an exosuit to reduce the metabolic effort in walking (Quinlivan et al., 2017). In the second synergy, hip and knee extensions show similarities in joint moment and power profiles during stance phase.

The gravity synergy can be used during movements in everyday life to support users with limitations in muscle force, when moving against gravity (for example getting up from a chair and going up the stairs), when moving with gravity (for example sitting on a chair), and to compensate gravity by increasing joint stiffness (standing).

The process of identifying the synergies is too conservative since it assumes a high-stiffness system, 100% of assistance delivered by the device, and it neglects the influence of the user and the biological muscles. Since a tensile actuator can only increase or reduce tendon length, but not both simultaneously, there can still be power generation and damping in coupled joints. This is due to the fact that the Myosuit is a bi-articular exomuscle that is added to the biological set of muscles. The hip and knee joints can use the applied forces to generate power—or dampen movements—in combination with the biological muscles, depending on the influence of external forces (i.e., gravity).

The rectus femoris provides evidence that positive and negative power can occur simultaneously in coupled joints. This bi-articular muscle connects the knee and hip joint and acts as both knee extensor and hip flexor. It is active during damping phases of the knee and power generation phases of the hip. The body exploits this behavior to use the maximum change in muscle length most efficiently (Elftman, 1966; Winter, 2009).

To provide the necessary anti-gravity support during walking, the contribution of the anti-gravity muscles to the joint torques have to be taken into account. Anderson et al. reported that the extensor muscles made the largest contribution to support, accounting for 50–95% of the vertical ground-reaction force generated in stance during normal walking. Gluteus Maximus, Vasti, Gluteus Medius/Minimus generated most of the muscular support (Anderson and Pandy, 2003). A consistent extensor pattern during stance phase was also shown by Winter: the support moment—defined as the algebraic sum of the joint moments of the lower limbs (Winter, 1984).

From a functional perspective, the Myosuit must prevent collapse during stance phase and allow the leg to swing freely forward during swing phase. To provide assistance to the hip and knee flexors, the passive ligaments must be able to compensate for portions of the weight of the limb segments and provide support during swing. The importance of the anti-gravity synergy of the hip and knee joint becomes even more apparent during movements where the potential energy of the center of mass (COM) changes significantly. This is for example the case in sitting transfers where the height of the COM changes significantly and the anti-gravity muscles have to provide extensive mechanical work to support the movement. This is why the Myosuit was first evaluated during sit-to-stand and stand-to-sit movements.

### 2.2. Design

**Figure 3:**
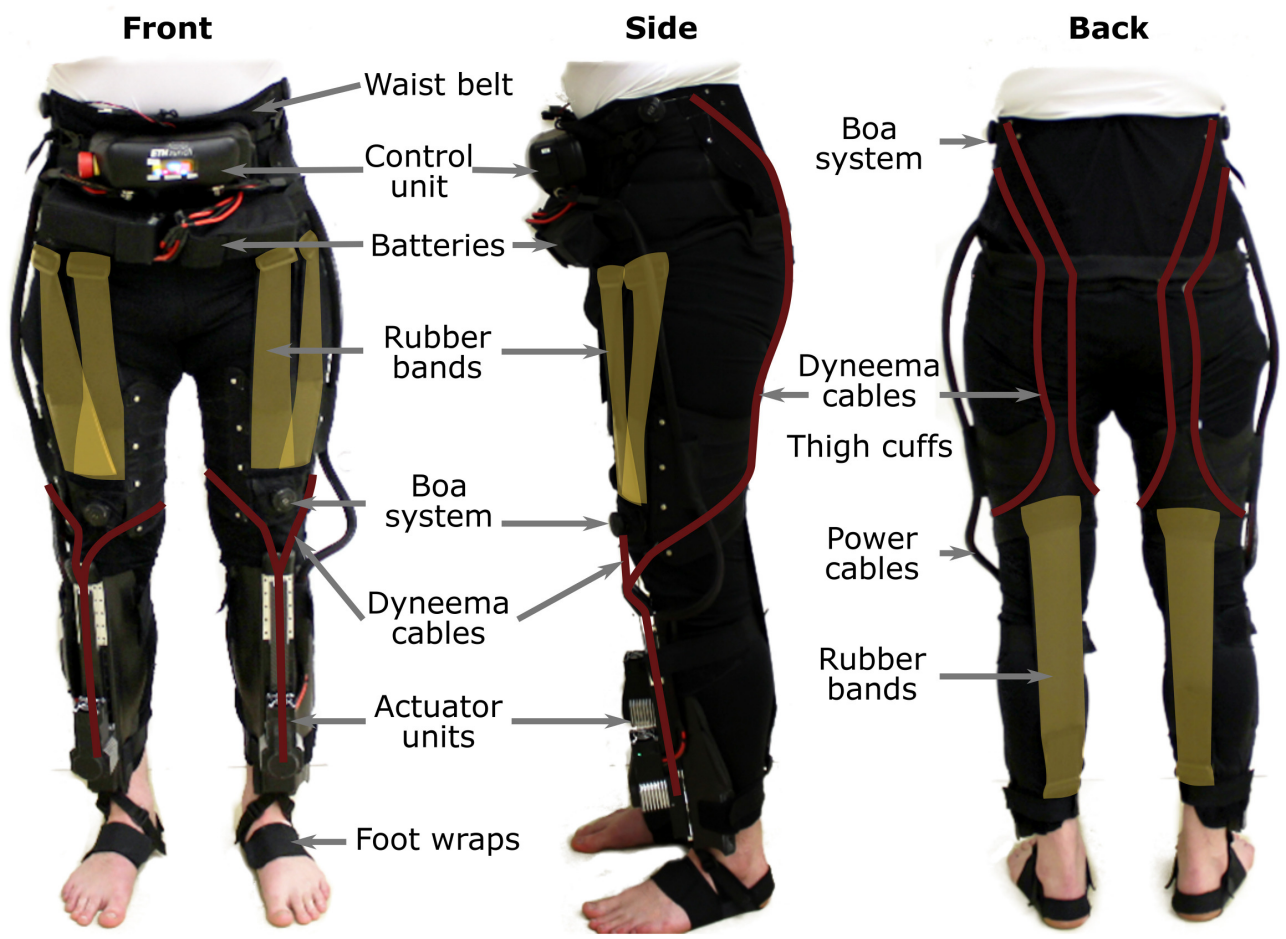
The Myosuit resembles a pair of pants that include a waist belt and thigh cuffs sewn onto a stretchable base material. One set of rubber bands, designed to aid with hip flexion, attach at the front of the waist belt and the front of the thigh cuffs. A second set of rubber bands, designed to aid with knee flexion, attach at the back of thigh cuffs and the base of the actuation unit. The waist belt and thigh cuffs can be adjusted to the user's body to ensure proper fit of the device and efficient transmission of forces. The waist belt houses the control unit and a set of batteries used to power the actuator units. Power cords connect the control unit to the actuators and provide the required power for the system. Dyneema cables, routed along the garment layer, define the actuation path of the Myosuit.

The Myosuit was designed to allow its user to sit comfortably in a wheelchair. Therefore, all rigid components (control unit, batteries, tendon actuators) are distributed across the body without limiting the ability to sit. The control unit and the batteries are mounted close to the COM (see Figure 3) and the tendon actuators are mounted distally at the shank. It is assumed that the COM is in front of the lumbosacral junction when the user is standing upright. The ligament layer compensates for the added distal mass. It is also expected that the user will only move slowly which keeps the influence of inertia low.

#### 2.2.1. Myosuit layers

The garment layer of the Myosuit resembles a pair of pants (1,380 g) and includes a waist belt and thigh cuffs sewn to a stretchable base material (Spandex). It also includes the attachment points for the ligament and the active layer. The integrated waist belt and thigh cuffs (Figure 3) are adjusted to the user's body to ensure a tight fit that minimizes slippage, and thus, allows consistent transmission of force to the biological joints. This is accomplished by a corset-like structure in both the cuffs and the waist belt.

Two Velcro flaps help close the waist belt on top of which the corset applies additional compression. The corset is tensioned on top of multiple polyethylene segments (2 mm thick) that support the lower back and increase the longitudinal stiffness. To minimize shear forces on the skin, the tendons are not attached directly to the polyethylene segments. Instead, a separate layer of nylon and webbing (polyester), placed above the segments, serves as the attachment point. Possible slippage due to tangential forces is limited to the segments. The shearing on top of the segments results in predominantly normal forces transmitted to the user's skin due to the conical shape of the waist. The waist belt prevents the downward migration of the garment layer when the tendon actuators are active.

At the thighs, cuffs equipped with a corset and Boa system (Boa Technology Inc.) help increase the level of compression of the soft tissue and the total stiffness of the Myosuit (Figure 3). Additionally, these cuffs prevent any upward shift of the garment layer and align the tendons relative to the knee.

Both the waist belt and thigh cuffs are made of inelastic Cordura-nylon-fabric (330 and 500 den), reinforced by layers of polyamide (1 mm) and a Cordura-nylon-Dacron-laminate (X-Pac VX21). The direct interface to the human body is partially cushioned by an inelastic 3D-spacer-mesh (polyester, 3 and 6 mm thick).

The active layer uses inelastic and abrasion-resistant tendons (Dyneema/UHMWPE, 1.2 mm diameter) to transmit the assistive forces to the user. The artificial tendons (Figure 3) are guided through the garment layer inside of Teflon (PTFE) tubes connecting the waist belt and the actuators. The tubes avoid small bending radii to reduce friction and use gaps between the tubes to allow for length adjustments of the actuation path i.e., when the Myosuit is active. The tendon actuators, which are part of the active layer, are secured by elastic bands wrapped around the shank and firmly anchored through foot wraps (32 g each) that prevent any upward movement when forces are applied (Figure 3). The actuation path extends from the waist belt (posterior) to the thigh (anterior) until it crosses the knee joint and connects to the tendon actuator (Figure 3). This posterior-anterior transition is done by guiding two artificial tendons symmetrically along the thigh, one lateral and one medial. The routing provides a moment arm at the hip of about 10–12 and 10 cm at the knee. The tendons are connected to the actuators by an adapter that helps distribute the forces equally (medial and lateral) when the system is actively pulling. The Boa system at the waist belt (Figure 3) is also part of the active layer and enables the user to adjust the initial tendon length by ±10 cm and to pretension the system.

The ligament layer uses passive elements to connect the thigh cuff and the waist belt (anteriorly) and the thigh cuff and the shank (posteriorly) at each leg. The passive elements (rubber bands) are stacks of different Thera-Bands that can be adjusted to achieve different strengths (Figure 3). The current setup uses two layers of Thera-Band Gold and two layers of Thera-Band Black that combine for a force of about 215 N for 30% of elongation. To minimize the shear forces applied to the user's skin, the ligament layer uses the garment layer in a similar way to the power layer. Additional nylon layers and webbing are used to allow possible slippage due to tangential forces on a separate layer on top of the user's skin.

#### 2.2.2. Tendon actuators

**Figure 4:**
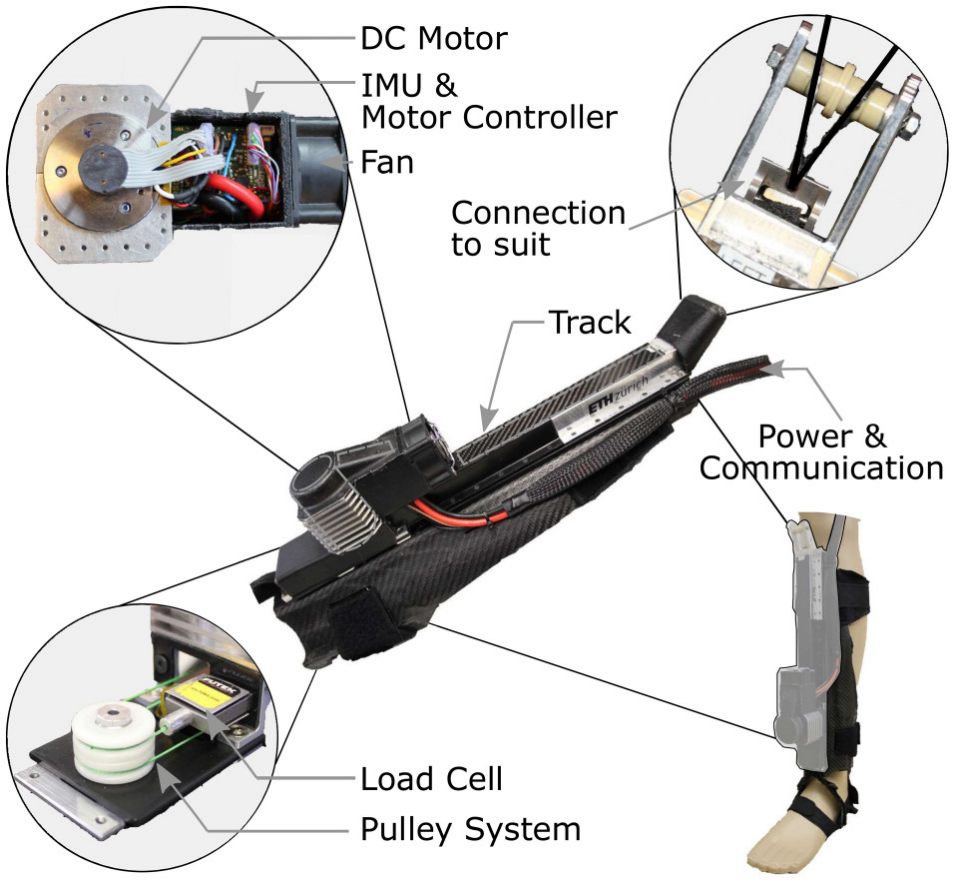
Tendon actuator. Two actuator units, each weighing 1,070 g, provide the assistive forces of the Myosuit. The actuator is placed on top of a carbon fiber shin-plate. Each unit incorporates a 70 W brushless DC motor coupled to an encoder. An inertial measurement unit, motor controller, and cooling fan are placed next to the motor with power and communication leads extending from the actuator to the central control unit. A Dyneema (0.6 mm) cable is fixed at the motor shaft (6 mm) and connects via a pulley system to the upper end of the actuator unit. At the upper end, the actuator cable is connected to the multiarticular cable connecting to the waist belt. The pulley system transmission ratio is 1:4. The unit allows for a maximum cable travel of 0.24 m.

Two actuator units, each weighing 1,070 g, provide the supportive forces of the Myosuit (Figure 4). The actuator is placed on top of a carbon fiber shin-plate. The design incorporates the artificial moment arm (*r*_*knee*_ 0.1 m) for the actuation of the knee (*r*_*knee*_ in Figure 2). Each unit incorporates a 70 W brushless DC motor (Maxon EC-i40) that is actively cooled by a fan. A Dyneema (0.6 mm) cable is fixed to the motor shaft (6 mm in diameter) and connects via a pulley system to the upper end of the actuator unit. The Dyneema cable is wrapped around the motor shaft multiple times when the system is active. Since the actuators are force controlled, there are no sudden jumps in force due to multiple wraps around the shaft and corresponding change of the effective diameter. The effective diameter cannot be measured or modeled exactly, and thus, there is no accurate position measurement embedded in the unit. It is for this reason that Myosuit's specific characteristics are reported in encoder count dependent values—whereby 2,048 counts correspond to 1 rotation of the motor shaft. At the upper end, the actuator cable is connected to the multi-articular tendons connecting to the waist belt. The pulley system transmission ratio is 1:4. The unit allows for a maximum cable travel of 0.24 m, which equals approximately 86,000 encoder counts.

The efficiency of the tendon actuators is 86% and the system's efficiency, including the textile layers, is 68%. Therefore, forces at the knee will always be higher than the forces applied to the hip because of the longer cable and increased friction. The efficiency of the tendon actuator was determined by comparing the commanded torque multiplied by angular velocity—the ideal performance—to the force at the tendon attachment adapter multiplied by the tendon velocity. The efficiency of the textile layers was determined in the same way, but on a previous version of the suit which used the same tendon routing and Teflon tubes.

The tendon actuators can deliver 435 N of force during continuous support of ADLs and are able to withstand forces up to 630 N for a short time (for example sudden collapse of the user). The maximum forces depend on the suit's stiffness and the available cable travel; the stiffer the Myosuit layers, the higher the maximum forces for are given cable travel. This is mainly because the maximum cable travel is partially used to compensate slack in the system and to compress soft tissue. The position bandwidth of the tendon actuators is 3.5 Hz. The actuator's bandwidth was tested by attaching it to a subject in a sitting position while performing a sine sweep (range of 0–20cm). For the closed-loop response of the controlled variable, the 3 db attenuation from the setpoint occurred at 3.5 Hz. The maximum forces achieved during this test were about 435 N. Since the Myosuit is designed for people that are having mobility problems, it is not expected that the suit has to react any faster than traversing almost full cable travel within 280 ms.

#### 2.2.3. Sensors

The Myosuit uses an array of motion sensors to estimate the user's movement intention, movement task, and current posture. Each actuator unit includes a force sensor (FUTEK, LSB200; maximum capacity of 445 N) to measure tendon forces and a motor encoder (Maxon motor, ENX16 EASY; 512 couns/turn) to measure the change in tendon length. The load measures the force in the pulley system before the change in gear ratio of 1:4 (Figure 4). The load cell signal is filtered by low-pass Butterworth filter (2nd order, cut-off 50 Hz, sample rate 1 kHz). There are three inertial measurement units (IMUs), one located in the control unit (STMicroelectronics, LSM9DS1), and two located on each shank (IMU; STMicroelectronics, LSM9DS0). The IMUs provide information on the acceleration (at 0.0012 m/s^2^ resolution) and rate of rotation (at 0.0175 deg/s resolution) of the trunk and shanks. The IMU data is transmitted through an I2C bus (400 kHz) and sampled at 100 Hz. A Kalman filter (process noise covariance = 0.001; measurement noise covariance = 0.03) is used to compensate for any drift in the tilt angle calculation for both the control unit near the COM and the shanks.

#### 2.2.4. Control unit

The Myosuit uses two ARM M4 micro controller units (MCU; NXP MK64FN1M0VLL12; 120 MHz, programming language C), one per leg, each running a FreeRTOS Kernel at 1 kHz. The motors are controlled by two servo drives (ELMO, Gold Twitter) that communicate with the MCUs using the CANOpen protocol. The control unit weighs 508 g.

The control unit is fixed at the front of the waist belt by Velcro and webbing straps. Below, two 22.2 V lithium polymer batteries (1,200 mAh each, 40C) are fixed using Velcro. The batteries are connected in series for an output voltage of 44.4 V. In case of emergency, the Myosuit can be switched off by an emergency button, located on the side of the control unit, that cuts the power between the batteries and the motors. Other security features include motor speed limits, cable force and length limits, motor temperature limits, and power-down mode (low battery voltage).

A button at the front of the control unit is used to switch between two assistance modes: anti-gravity support—when moving against gravity—and gravity support—when moving with gravity. A second button is used to switch to a transparent mode where the user can move with minimal resistance on the cable (tendon tension 5 N).

The system is fully self-contained and does not need any external cable connected during operation. To synchronize the Myosuit with external systems, such as motion capture equipment, an input connection is available to read a triggering signal. Myosuit data (encoder counts, tendon force, raw IMU data, trunk angle, shank angle, synchronization input trigger) of each leg is sent over Bluetooth to a computer at 100 Hz for data logging.

### 2.3. Closed-loop control algorithm

**Figure 5:**
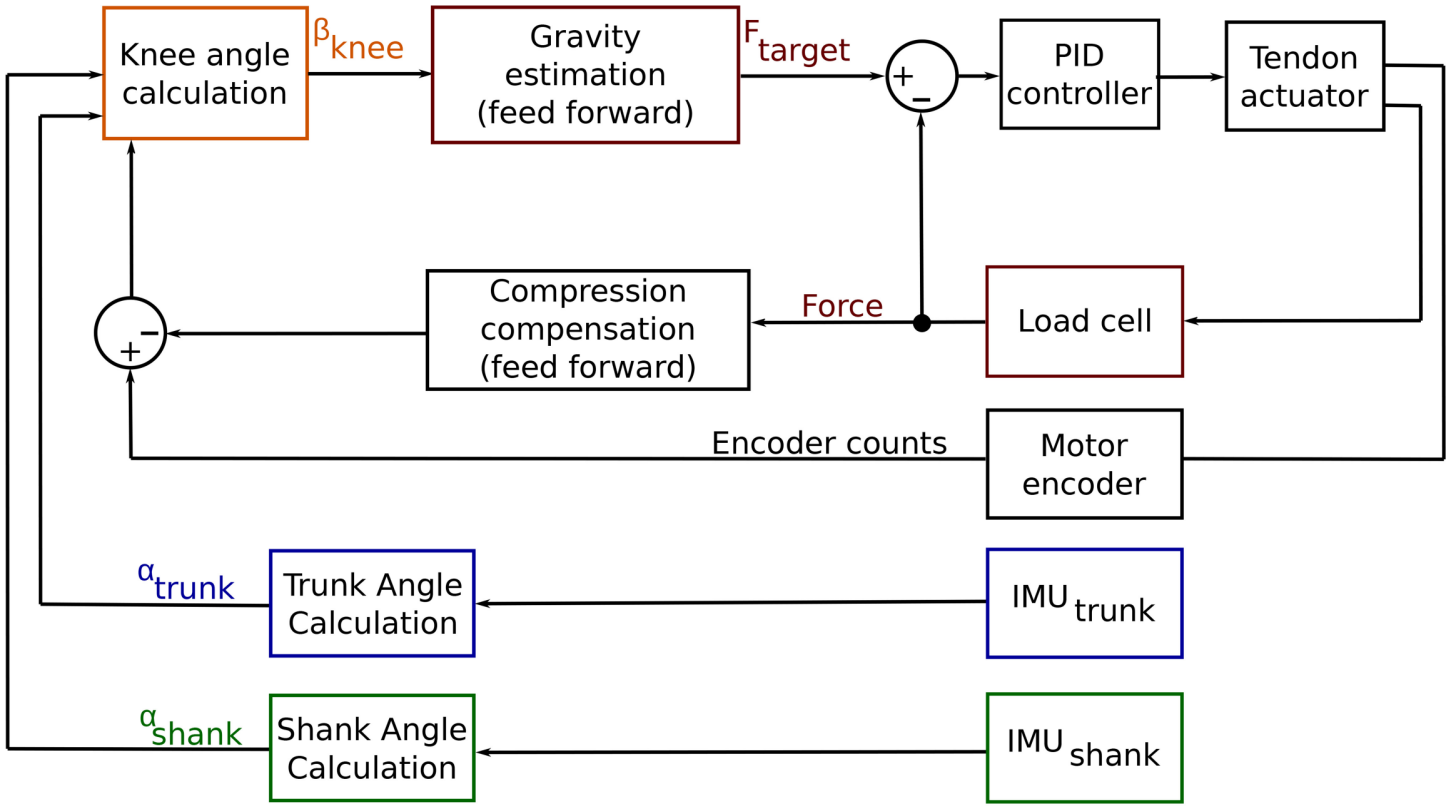
Control chart for the Myosuit. The goal of the Myosuit is to provide its user with anti-gravity support. This approach aims to support the extensor torques at the knee and the hip joint. The controller is based on a virtual leg that connects the hip and ankle joints. The length of the virtual leg scales with the knee angle β_*knee*_. The force delivered to the user is defined to provide the highest moments at a knee flexion angle of 80° and decreases as the angle increases. Controller inputs are the shank angle, α_*shank*_, trunk angle, α_*trunk*_, and the length of the tendon. Using these inputs, the knee angle, β_*knee*_, is calculated in real-time and used to adjust the level of force delivered to the user based on the user's current posture. The target force *F*_*target*_ is then fed into a PID controller that is set to the desired force level.

The Myosuit provides anti-gravity assistance (with and against gravity) by supporting the extensor torques at the knee and the hip joint. The controller uses an array of motion and forces sensors to continuously estimate the user's movement intentions and posture at 100 Hz. Figure 5 shows the simplified control chart of the control algorithm. The system calculates the influence of gravity on the user's joints—knee and hip—and compensates for this external force by modulating the tendon forces and stiffness accordingly. Controller inputs are the shank angle, α_*shank*_, trunk angle, α_*trunk*_, and the length of the tendon. Using these inputs, the knee angle, β_*knee*_, is calculated in real-time and used to adjust the level of force delivered to the user based on the user's current posture—a full derivation is given below. The target force *F*_*target*_ is then fed into a PID controller that sets the final force level. The anti-gravity concept can be used with minimal adaptation across the targeted ADLs (ground-level walking, walking up and down slopes, stair ascend and descent, sit-to-stand, and stand-to-sit transfers). This article focuses on anti-gravity assistance for sit-to-stand and stand-to-sit transitions.

#### 2.3.1. Anti-gravity control against the force of gravity

**Figure 6:**
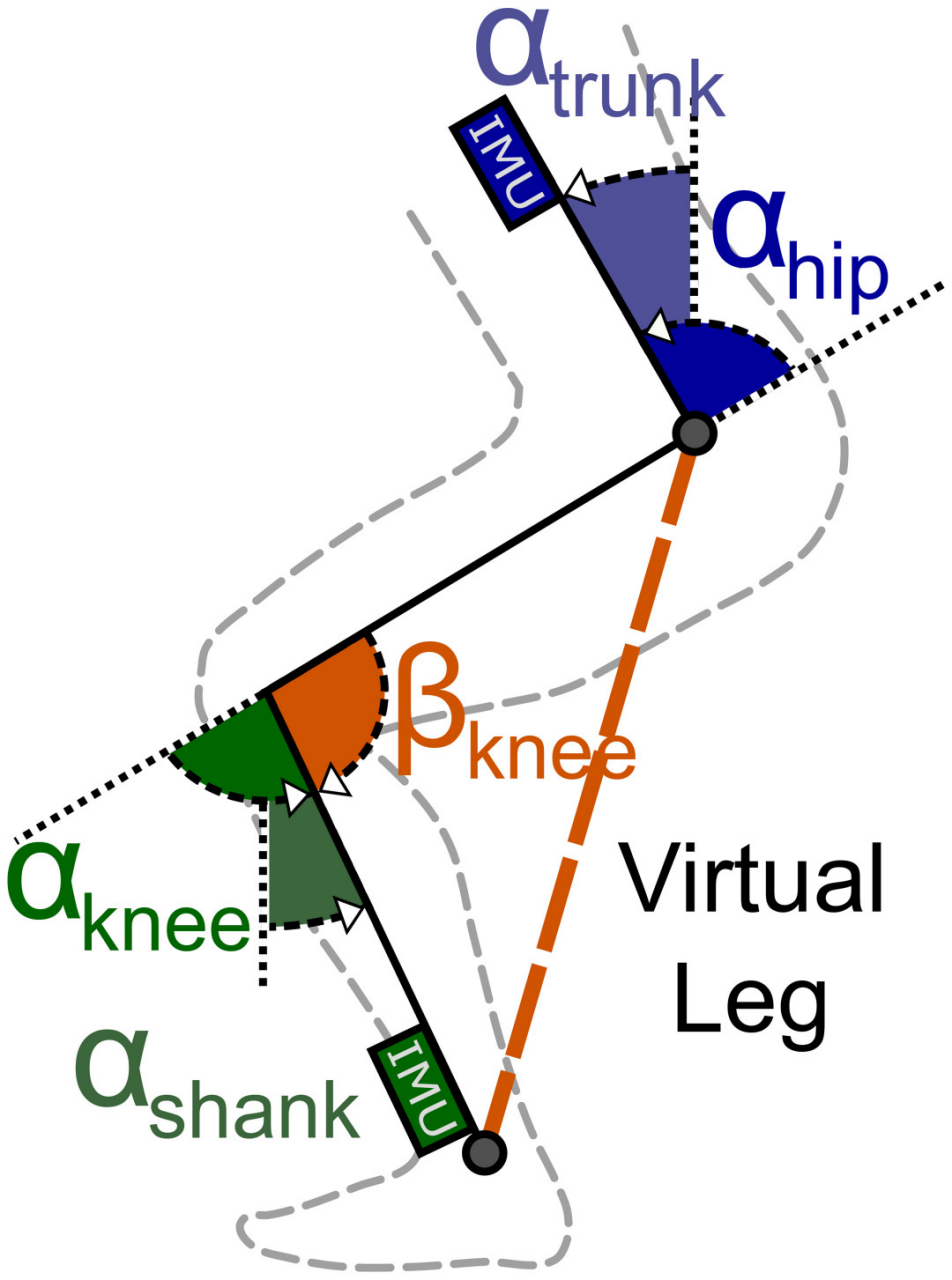
Virtual leg model. IMUs placed at the shank and trunk measure the shank (α_*shank*_) and hip (α_*hip*_) angles. The motor encoder measures the length of the cable routed along the leg. These parameters are used to compute the knee angle β_*knee*_. This angle scales with the virtual leg length that is used to scale the anti-gravity assistance forces.

The anti-gravity control provides an assistive extension moment at the knee and hip joints. The controller is based on a virtual leg that connects the hip and ankle joint. The virtual leg length scales as a function of the knee angle β_*knee*_ (Figure 6). The calculated knee angle is used to adjust the assistive forces dependent on the maximum torque at a knee flexion angle of 80°. As the angle increases, the virtual leg becomes more vertical—and therefore more stable—and the forces is thus reduced.

The knee angle, β_*knee*_, is calculated in real-time using the measurements from the system's IMUs and motor encoders. The shank angle, α_*shank*_, is determined using the IMU at the actuator unit. The trunk angle, α_*trunk*_, is determined using the IMU at the control unit. The beta angle is then defined as the angle between the thigh (direct connection between hip and knee joint) and a vertical line perpendicular to the ground (see Figure 6). The following trigonometric relation exists between these angles:

(1) $$\alpha_{knee}-\alpha_{shank}=\alpha_{hip}-\alpha_{trunk}$$

Since both α_*knee*_ and α_*hip*_ are unknown, an additional parameter — motor encoder counts (*EC*)—is used to solve the equation for α_*knee*_ in Equation (1). A series of experiments was conducted to characterize the relation between encoder counts and the movements of the knee and hip joints. The relation between encoder counts and the α_*knee*_ angle, *EC*_*knee*_, was measured by flexing and extending the knee joint from 80 ° to a fully extended position while sitting on an elevated platform. The relation between encoder counts and the α_*hip*_ angle, *EC*_*hip*_, was measured by leaning the trunk forward against a rail while keeping the legs straight (fully extended knee joint).

To compensate for changing encoder counts due to tissue compression, the Myosuit's stiffness was measured. The suit stiffness is the relation between encoder counts, *EC*_*force*_, and the tendon force, *F*_*tendon*_, under isometric conditions. For this measurement, the subject remained in a sitting posture—with both the knee and hip angles held constant—while the tendon actuator cycled 23 times from a minimal value of 5 N to a maximum value of 350 N. The measurements were taken while sitting because the system's forces, and therefore the effect of tissue compression, will be highest in this posture. The tendon force of 350 N was chosen because up to this force it was possible for the subject to withstand the forces without any movement.

The change in knee angle (β_*knee*_) was calculated using the effective change in encoder counts (*EC*_*effective*_). This is the corrected number of encoder counts based on the stiffness of the Myosuit (*EC*_*force*_). The *EC*_*effective*_ parameter is defined as the difference between the encoder counts measured by the encoder (*EC*_*total*_) and the estimated encoder counts needed to compress the soft tissue by the applied force (*EC*_*force*_).

(2) $$EC_{effective}=EC_{total}-EC_{force}$$

where:

(3) $$EC_{force}=\frac{F_{cable}}{m_{force}},$$

and *EC*_*effective*_ is the sum of the encoder counts due to the change of the hip and the knee angle; with *m*_*force*_ being the relation between the tendon force and the change in encoder counts (stiffness of the Myosuit).

(4) $$EC_{effective}=EC_{hip}+EC_{knee}$$

By replacing *EC*_*hip*_ and *EC*_*knee*_ in Equation (4) we obtain:

(5) $$EC_{effective}=\frac{\alpha_{hip}}{m_{hip}}+\frac{\alpha_{knee}}{m_{knee}}$$

Where *m*_*hip*_ is the relation between the change in hip angle and encoder counts, and *m*_*knee*_ is the relation between the change in knee angle and encoder counts. Finally, Equations (1, 5) can be combined to calculate β_*knee*_:

(6) $$\beta_{knee}=180-\frac{m_{knee}\cdot (EC_{effective}\cdot m_{hip}+\alpha_{shank}-\alpha_{trunk})}{m_{hip}+m_{knee}}$$

The assistive forces were scaled such that they were lowest in the standing posture ($\beta_{knee}\approx 180^{{}^{\circ}},\;F_{Tendon}=5N$) and highest in the sitting posture ($\beta_{knee}\approx 100^{{}^{\circ}},\;F_{Tendon}=350N$). To validate the relation between moment and angle of the hip and knee joint, knee and hip moments were measured during sitting transfers using a motion capture system (Vicon, 10-camera system, Vicon Motion Systems Ltd.) and the ground reaction forces using one force plate (Kistler force plate 9260AA, Kistler Holding AG) per leg. The assistance for the sit-to-stand transition was automatically switched on when the tilt angle of the COM exceeded 0° during sitting.

#### 2.3.2. Anti-gravity control with the force of gravity

For stand-to-sit transition the anti-gravity control is used similarly to the method introduced before by using the virtual leg calculations β_*knee*_. However, the level of assistance was scaled down to allow the subject to sit and not cause locking of the joints; that is, if the force level was the same as during sit-to-stand, the subject was unable to sit and instead had to actively work against the system to flex the knee joint (i.e., contract the hamstrings to overcome the Myosuit's force). A scaling factor of 35% of the sit-to-stand forces was found to work well in pilot tests of the device. A fixed knee angle of 115° was defined to switch off the assistance shortly before chair contact. This was done to prevent large forces from being applied to the user while in the sitting position.

### 2.4. Experimental protocol

To test the control concept of the Myosuit, a 28-year-old subject (height: 1.8 m; mass: 80 kg) performed ten sit-to-stand and stand-to-sit transitions under non-assisted and assisted conditions (Supplementary Material). In the non-assisted condition, a constant tendon force of 5 N was set in order to have a taut tendon. In the assisted condition, the tendon forces were defined by the anti-gravity controller previously described. The forces were scaled depending on the transition performed. For sit-to-stand transitions, the scaling value was set to 1; for stand-to-sit, the scaling value was set to 0.35. That is, the tendon forces during stand-to-sit were set to 35% of the sit-to-stand forces.

**Figure 7:**
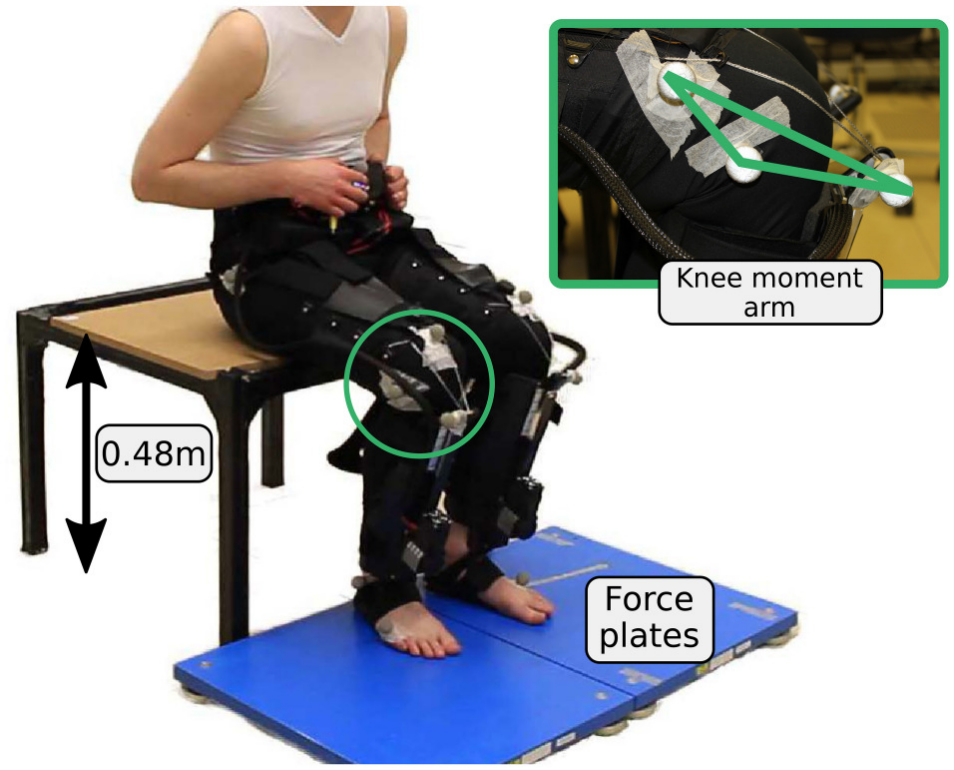
Experimental setup for the sitting transitions. Two force plates are used to evaluate ground reaction forces.

Between transitions, the subject was instructed to either stand or sit upright. Transitions were initiated by a metronome with 12 s intervals. At the beginning of each experiment, the Myosuit, force plates, and the motion capture system were synchronized with a 3 V trigger signal. The height of the chair was selected to match the mean wheelchair height of 0.48 m which is equal to DIN 18040-1, the public restroom standard (Figure 7).

Joint kinematics were measured at 200 Hz using the Vicon motion capture system. Ground reaction forces were measured at 1 kHz using one Kistler force plate (Kistler force plate 9260AA, Kistler Holding AG) per leg. Muscle myoelectric activity was measured at 1 kHz for the hip extensor gluteus maximus and the knee extensor vastus lateralis using a wireless Noraxon system (Telemyo DTS). Gluteus maximus and the quadriceps are the main anti-gravity muscles and the main contributors to the sit-to-stand transitions (Roebroeck et al., 1994). Since the Myosuit requires a tight fit, it was only possible to measure gluteus maximus and vastus lateralis reliably with the current version of the Myosuit layers.

The study was approved by the institutional ethics review board of ETH Zurich. The participant provided written informed consent before participation.

### 2.5. Data analysis

#### 2.5.1. Kinematics and kinetics

Marker and force data were filtered using a zero-lag Butterworth filter (fourth order). A cutoff frequency of 6 Hz was used for the marker; and 10 Hz for the force data. After calculating joint angles and angular velocities, the kinematic data was filtered offline using a zero-lag Butterworth filter (second-order) with a cutoff frequency of 10 Hz.

The hip angle was calculated between a marker placed centered on top of the control unit, the trochanter major, and an assumed center of rotation at the knee (2 cm proximal of the joint space on the lateral femoral condyle). The knee angle was calculated as the angle between the line connecting the greater trochanter major, the rotational knee point and the line connecting the rotational knee point and the lateral malleolus. Angular velocities and accelerations of the joints were calculated by numerical differentiation and were multiplied by the joint moment to obtain joint powers. Joint moments were determined using a static approach (Günther et al., 2003). The knee moment of the Myosuit was calculated based on the knee lever-arm and the tendon force. To calculate the knee lever-arm, reflective markers were placed where the tendons exit the Teflon tubes and where the tendon connects to the actuator (Figure 7). The Myosuit knee power was calculated by multiplying the Myosuit moment and the knee angular velocity. Moments were normalized to individual subject body mass.

#### 2.5.2. Muscle activity

EMG data was band-pass filtered (fourth order Butterworth, cut-off 20–450 Hz), rectified, and low-pass filtered (fourth order Butterworth, cut-off 6 Hz). The mean of up to ten repetitions was normalized for each muscle to the total peak value from the four conditions: sit-to-stand, stand-to-sit, both with and without assistance. To reduce the offset, the minimum of the four conditions was identified and it was then subtracted from all conditions.

#### 2.5.3. Transition identification

**Figure 8:**
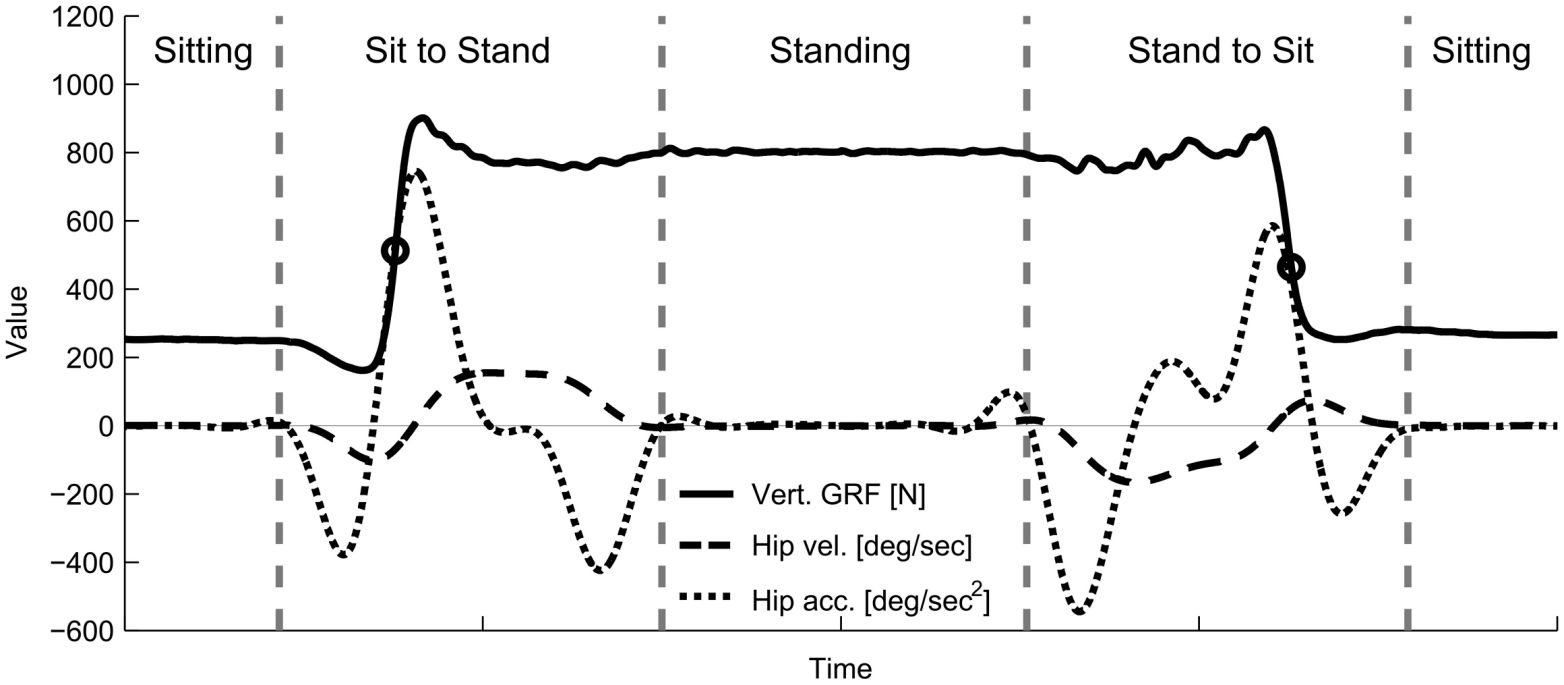
Example hip kinematics and ground reaction forces used to segment the recorded data offline for the analysis. Total ground reaction forces, hip angular velocity, and hip angular acceleration for the sit-to-stand and stand-to-sit transitions were measured using the force plates and the motion capture system. Circles indicate the time of the maximum change in vertical ground reaction forces. Starting from this event, hip kinematics were used to identify the beginning and the end of each transition (vertical line).

The sit-to-stand and stand-to-sit transitions were cut and normalized for the subject. To cut and normalize the data it was necessary to identify the point where the gradient of the ground reaction forces reaches its maximum for both transitions (Figure 8). The beginning of sit-to-stand was identified by using the maximum change during the increase in ground reaction forces (sum of both legs). By using previous samples, the time was identified were hip angular velocity and hip angular acceleration became more negative than −10 deg/s and −10 deg/s^2^ (sum of both legs), respectively. The end of the sit-to-stand transition was identified by using the next samples to find the time when the hip angular acceleration became more negative than −10 deg/s^2^ and the hip angular velocity fell below 10 deg/s. The stand-to-sit transition was identified using the maximum change during decreasing ground reaction forces following the same method explained above.

The time was normalized for the first part of each transition (from start to the point of the maximum force gradient). In addition, the second part was normalized from the point of maximum change in force until the end of the transition. The length of the first and the second part was set by determining the average time for the first and second part for all transitions before time normalization. This method to identify transition was used offline after data collection.

## 3. Results

### 3.1. Compression compensation

For the relations between joint angles and motor rotations we report the more standard measure of angles: radians. Note that our internal calculation relied on the encoder counts (EC) as described in the Methods section of the article. The relation is given by: 1*EC* = 2π/2048*EC*. We acknowledge that EC is not a standard engineering unit nor a hardware independent variable. However, it is important to point out that the tendon (cable) is wrapped around the motor without being guided. This means the change in winch diameter due to multiple cable wraps is not known, and thus, the exact relation between tendon length and motor rotation is not known either. Since the system does not rely on internal cable position measurement and purely on the tendon force, this design does not affect the usability of the system.

**Figure 9:**
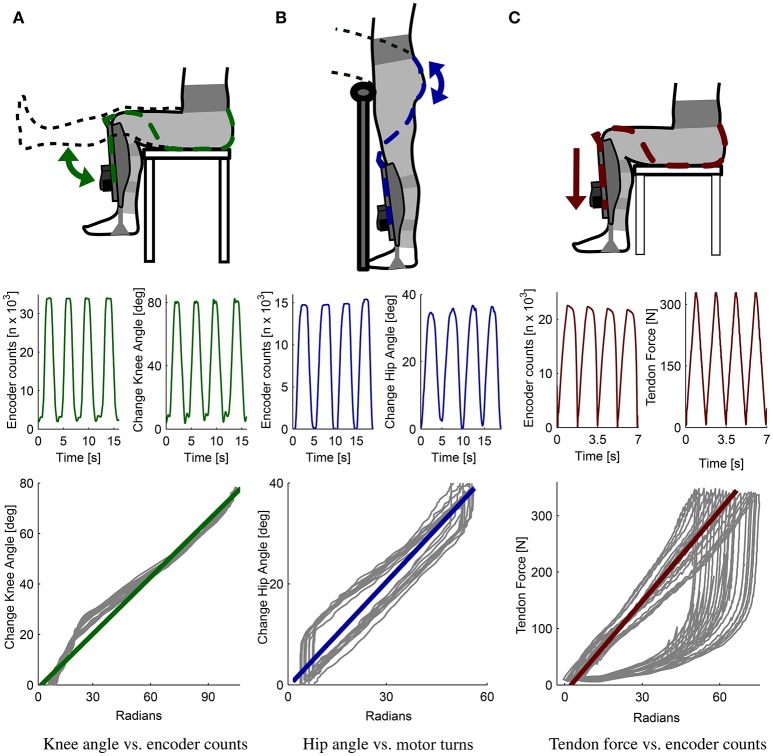
Myosuit characterization. Relation between movements of the knee and hip angles and changes in tendon length (measured as encoder counts). Separate measurements were conducted for each joint to define the relation between the change in knee angle and the encoder counts **(A)** and the hip angle and the encoder counts **(B)**. For the knee angle, the relation obtained is 0.75 deg/rad; for the hip angle, the relation is 0.68 deg/rad. The relation between the cable force and the encoder counts was also determined experimentally **(C)** and gives us a measure of the suit's stiffness at 5.28 N/rad. These three relations were used to determine the knee angle β_*knee*_ in real-time using the virtual leg controller.

The relation between the change in knee angle and encoder counts (*m*_*knee*_) was approximated as linear with a slope of: 0.75 deg/rad (Figure 9A; bottom row). The relation between the change in hip angle and encoder counts (*m*_*hip*_) was approximated as linear with a slope of: 0.68 deg/rad (Figure 9B; bottom row). The relation between the force on the tendon and the change in encoder counts (stiffness of the Myosuit), under the isometric condition, was approximated as linear with a slope of: *EC*_*force*_ = 5.28 N/rad (Figure 9C; bottom row) which approximately corresponds to 5,400 N/m. This curve shows a distinct hysteresis that the linearization of the loading phase cannot account for. However, it was found that this simplification still showed acceptable estimates for β_*knee*_.

**Figure 10:**
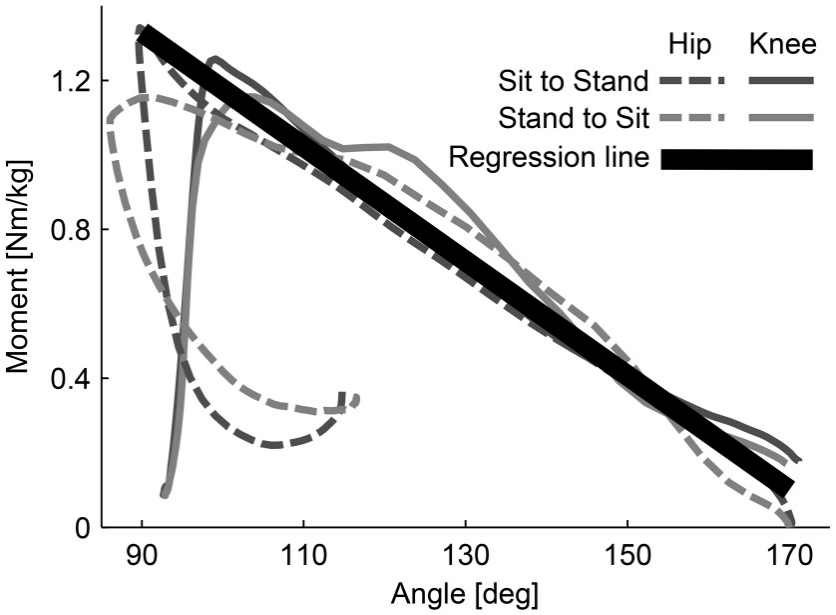
Moment-angle curve of hip and knee joints during sitting transfers. The curves were experimentally determined. The relation (regression line) is used in the controller of the Myosuit. When forces are actively applied during movements with and against gravity, the relationship between hip and knee angles is assumed to be linear. This relation is used to scale the assistive forces of the Myosuit.

For the relation between knee and hip moments and knee and hip angles, a linear relation between angles and moments from 90 to 170° was obtained (Figure 10). This linear relationship was approximately the same for transfers with and against gravity. Scaling the forces linearly dependent on the virtual leg will apply considerable and biological relevant torques to both the hip and knee joint. Since the Myosuit does not provide 100% of the biological torque, the forces were scaled linearly to the maximum continuous force during ADLs (435 N) and the lowest possible force (5 N; non-assist mode). The range of angles chosen (80 to 180°) to scale the assistive forces was slightly bigger then the one measured in Figure 10. This is the maximum range a user is expected to use in the suit during everyday life.

The following linear equation describes this behavior accounting for the artificial moment arm *r*_*knee*_ = 0.1 (Figure 2):

(7) $$Force=\frac{-0.43125\cdot \beta_{knee}+78.125}{0.1}$$

### 3.2. Posture based anti-gravity control

**Figure 11:**
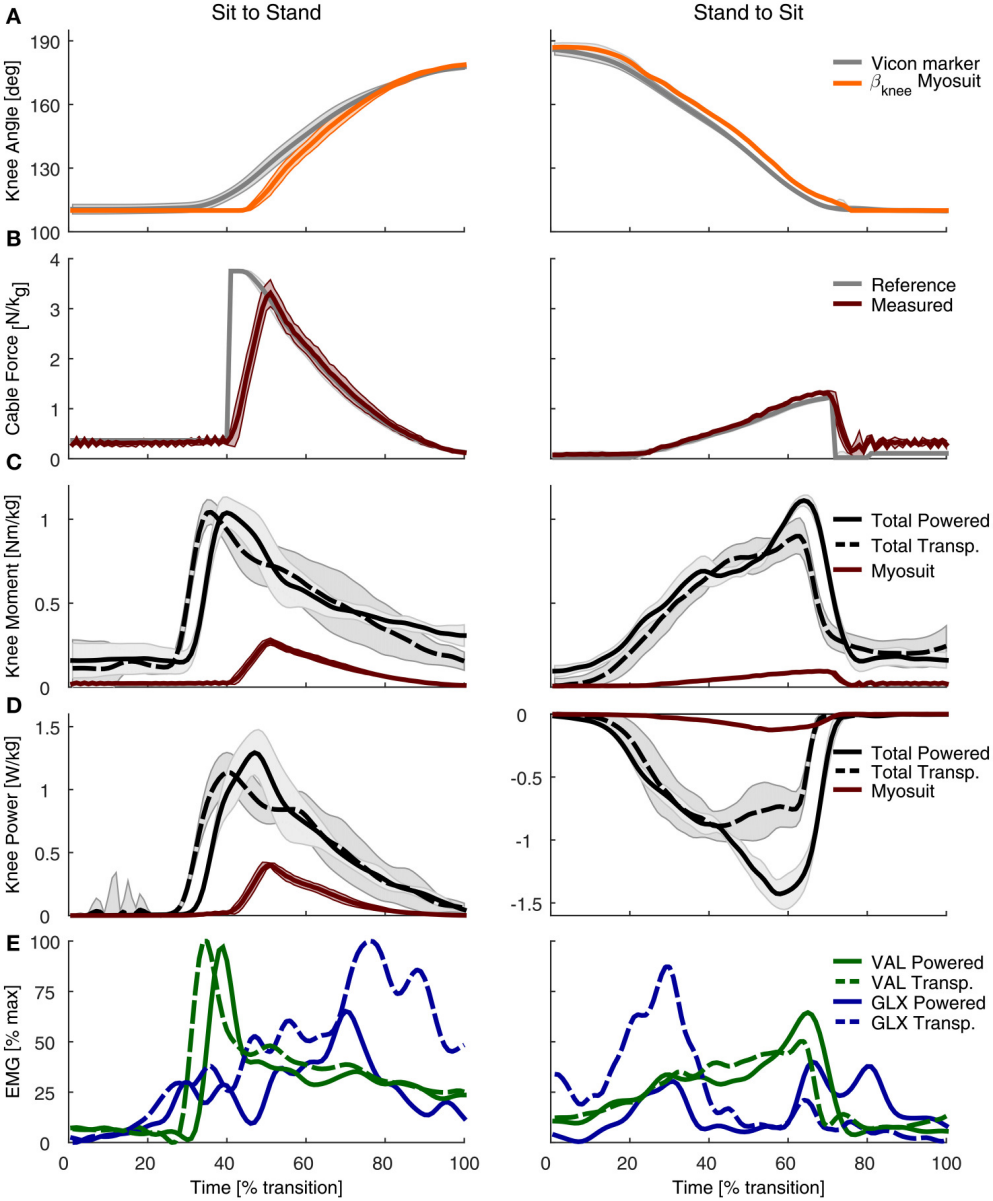
Experimental evaluation of the Myosuit during sit-to-stand (left) and stand-to-sit (right) transitions. **(A)** The β_*knee*_ estimation from the Myosuit's controller is shown alongside the value measured with the motion capture system. The estimate from the Myosuit is delayed, on average, by 11.48% relative to the motion capture measurements. The flat line at 110° is due to a safety feature designed to prevent large forces from being applied while the subject is in a sitting posture. **(B)** Desired tendon force (gray) based on the calculated β_*knee*_ and the actual tendon force as measured with the load cell (red). **(C)** Total knee moment based on motion capture data and ground reaction forces for the powered condition (solid black) and the transparent mode (dashed black). The knee moment of the Myosuit, based on tendon force and the knee lever arm, is shown in red. **(D)** Total knee power based on motion capture data and ground reaction forces for the non-assisted (dashed) and assisted (solid) conditions. The Myosuit power, shown in red, can be delivered for both sit-to-stand and stand-to-sit movements. **(E)** EMG recordings of the gluteus maximus (GLX, blue) and vastus lateralis (VAL, green) for the non-assisted (dotted) and assisted (solid) conditions.

The estimated knee angle, based on the virtual leg model, was used successfully to grade the level of gravity support provided to the user (Figure 11). During sit-to-stand, the estimated knee angle (β_*knee*_) lagged the measurement from the motion capture system by an average of 11.48% at the onset of the movement. This lag decreased to zero as the subject reached the standing posture (Figure 11A). During stand-to-sit, the estimated β_*knee*_ lagged the motion capture measure by 4.1%; this lag was present through the movement. The knee angle, as estimated by the Myosuit, had a minimum set to 110° due to a safety measure designed to prevent the controller from applying large forces while the subject was in the seated position. At this angle, only pre-tension forces of around 30 N were acting on the subject.

The performance of the controller was characterized by its ability to track the desired tendon force (Figure 11B). While the subject was in a sitting position, the tendon force properly tracked the desired pre-tension force of 30 N. Once the sit-to-stand movement started, the peak tendon force lagged the peak reference force by 11.2% and was lower by 35.72 N. During standing, the force on the tendon was tracked properly at the minimum value set to maintain the tendon taut: 5 N. During stand-to-sit, the largest difference in tendon force occurred toward the end of the movement—close to the sitting position when the forces were the largest—and was, on average, higher by 9.84 N. The transition times that correspond to 0–100% of the movement varied from 1.5 to 1.9 s.

### 3.3. Joint kinematics and kinetics

During sit-to-stand, the Myosuit delivered about 26% of the peak moment (0.27 Nm/kg) in the assisted condition relative to the non-assisted condition (1.04 Nm/kg) (Figure 11C). For stand-to-sit transitions a peak moment of about 0.1 Nm/kg was provided. This corresponds to about 11% of the peak total moment of the transparent condition (0.9 Nm/kg). Prior to the onset of the sit-to-stand transition, there was a time-related offset between the non-assisted and assisted conditions of 3.9%. During the stand-to-sit transition, there was an increase of the total knee moment in the assisted condition as the subject reached the sitting position.

The Myosuit provided about 35% of the knee peak power of the transparent mode (0.4 of 1.14 W/kg) during the sit-to-stand transition (Figure 11D). For the stand-to-sit transfer, about 15% of the peak power was provided by the Myosuit in the assisted condition when compared to the non-assisted condition (−0.13 of −0.89 W/kg). Peak power of the assisted condition in the stand-to-sit transfer was higher when compared to the unassisted condition.

### 3.4. Muscle activity

**Figure A1:**
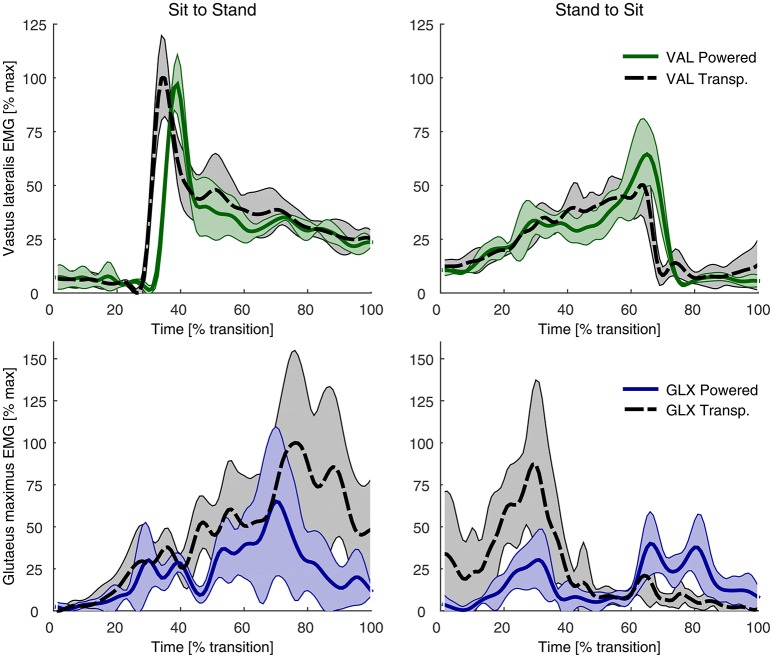
EMG recordings of the gluteus maximus (GLX, blue) and vastus lateralis (VAL, green) for the non-assisted (dotted) and assisted (solid) conditions.

For both sitting transfers, there was a general decrease in the muscle activation of the gluteus maximus (GLX). This reduction was present across the sit-to-stand transition, especially toward the end of the sit-to-stand transition. For the stand-to-sit transition, the reduction was largest at 30% of the transition where it was reduced by about 60%. Two additional muscle activity peaks in the gluteus maximus were present during assistance, at about 60–90% of the transition. These peaks were not present in the non-assisted condition. For the vastus lateralis (VAL), there were no clear differences in the magnitude of the muscle activity between the two conditions. During sit-to-stand, there was a lag in the onset of muscle activity, for both the gluteus maximus (GLX) and vastus lateralis (VAL), during the assisted condition (Figure 11E, Figure A1).

## 4. Discussion

The Myosuit is a lightweight and untethered wearable robotic device that uses a bi-articular design to counteract gravity by actively supporting hip and knee extension. In this article, we showed how this design can provide positive power to assist sit-to-stand transfers and negative power to support stand-to-sit transfers. The Myosuit aims at reducing the user's physical effort, quantified in this study by the changes in muscle activity of the knee extensor vastus lateralis and the hip extensor gluteus maximus. Instead of relying on predefined position-based triggers, the assistance concept presented uses a force-based approach that is related to the user's current posture. An initial test showed that the proposed control approach properly estimates the user's posture and intention in sitting transfers. Specifically the knee angle could be estimated by using the exosuit's tendons and linearized suit stiffness. Based on the knee angle, the Myosuit delivered positive and negative power to the user, resulting in up to 26% of the natural knee moment and up to 35% of the knee power. As a result, muscle activity of the hip extensor (gluteus maximus) was reduced in both sit-to-stand and stand-to-sit transfers. The Myosuit's novel approach to assistance using a bi-articular architecture, in combination with the posture-based force controller, can effectively assist its users in gravity-intensive ADLs, such as sitting transfers.

### 4.1. Compression compensation and knee angle estimation

A key component of working with a soft, textile-based structure is the optimization of the transmission of power from the robot to the user. For the Myosuit, the main inefficiencies arise from the friction between the tendons and the garment layer and from the compression of the user's tissue due to the tendon's tension. These effects are illustrated in Figure 9C where the motor winds close to 6 cm of tendon (≈20,000 encoder counts) to reach 324 N in an isometric condition. While in the loading phase the behavior is primarily linear, the unloading phase presents a clear non-linear relation between the force on the tendon and the winding of the motor. This unloading phase exhibits hysteresis and is characterized by decompression of the user's tissue and by the presence of friction between the tendon and the garment layer.

An unexpected finding was that only the loading phase of the curve had to be taken into account to correct for the Myosuit's stiffness. During initial tests, both the loading and unloading phases of the curve were taken into account when correcting the encoder counts. However, this led to an offset in the encoder counts—and an error in the estimation of β_*knee*_—during actual movements. Instead, if only the loading phase of the curve was used, the estimation of β_*knee*_ was far more accurate. This is likely due to the increased stiffness of the muscles when a movement is executed, as opposed to the isometric condition used for characterization where the muscles were inactive. Characterization of the Myosuit's stiffness during movements is not feasible since delivering force loading/unloading patterns in non-isometric conditions will result in the user changing posture.

The ligament layer can also affect the transmission of joint torques to the user. Although, it was inactive in this study—sitting transfers do not require assistance of hip or knee flexion—, other activities of daily life (e.g., walking) involve active flexion of the hip and knee. By engaging the ligament layer, the maximum joint torques in extension will be reduced. That is: *torque*_*net*_ = *torque*_*activeLayer*_ − *torque*_*ligamentLayer*_; where the *torque*_*ligamentLayer*_ is dependent on the joint's posture and the moment arm of the ligament layer *r*_*joint*_*ligament*_ (see Figure 2). Fortunately, the decrease in net torque is accompanied by an increase in joint stiffness, similar to a co-contraction of antagonistic muscles, which translates into increased stability for the user.

### 4.2. Posture based anti-gravity control

In a first experiment, sit-to-stand and stand-to-sit transitions were successfully assisted by the anti-gravity controller used by the Myosuit. The assistance concept uses a bi-articular structure to assist with knee and hip extension. The controller was able to identify the knee angle and scale the assistive forces accordingly. Compared to the change of angle detected by the motion capture system, the Myosuit's estimate showed a lag in detection. This might be due to inability of the system to detect changes in cable length without assistance because of its compliant structure. For safety reasons the minimal knee angle was set to 110° for the experiment. The lag could also be due to the fact that the system did not calculate the joint angle correctly and estimated a smaller angle than 110°. The anti-gravity support was triggered if a specific COM angle was exceeded. This simple trigger to switch on the anti-gravity assistance may have been set too late. A clear delay can be seen in the moment, power, and EMG plots. This needs to be validated in a further study.

The assistance applied resulted in 26% of the biological peak of knee torque during sit-to-stand transfer. The initial scaling of the maximum force (435 N) was set for an angle of 80°. In this case the minimum angle was 110° which resulted in a lower desired force (about 306 N). In addition to the late onset of force, the ramping up of force by the tendon actuator was set too low; this led to the lag in force relative to the desired setpoint. In this test, this lag led to a significant reduction in the maximum peak force. Nevertheless, the Myosuit was able to deliver about 35% of the peak power during sit-to-stand movements.

The power curves show an increase in power compared to the non-assisted condition. This is likely due to the increase in speed while standing up. While the vastus lateralis did not show a reduction in muscle activity, the peak EMG activity occurred at the onset of assistance. At this time, however, the Myosuit was not applying significant forces to support the movement. Applying the assistive forces earlier in the transition will likely lead to reduction of the muscle activity at the vastus lateralis.

On the other hand, the muscle activity of the gluteus maximus was reduced during the assisted condition reduction in the sit-to-stand transfer. This indicated that the forces, which are based on the knee, and not the hip angle, apply assistive forces that are biologically relevant and can assist the function of the hip extensors.

The applied forces resulted in 11% of the biological peak knee torque and 15% of the peak knee power during stand-to-sit transfer. The angle estimation shows a slight delay, but it is able to track the change of the angle reliably. An increase in peak moment and negative peak power can be seen before the Myosuit changes in the non-assist mode. This happened before the user touched the chair since the assistance was turned off based on a fixed angle. The sudden release of the force led to the user performing a movement that was less controlled when sitting; this effect likely affected some of the results. For example, due to a harder landing on the chair, the EMG of the gluteus maximus muscle seemed to have increased at 60–90% of the transition. This effect on the EMG reading might be a movement artifact caused by the less-controlled landing on the chair.

Immediately before the assistance was switched off, there was an increase in the EMG signal of the vastus lateralis. This was likely the result of a behavioral or learned muscle activation since the user performed multiple repetitions and the assistance was always turned off at the same knee angle. Thus the user may have developed this strategy to prevent a hard impact on the chair. To improve the stand-to-sit transition, the anti-gravity control should be changed from using a constant factor for scaling and instead include a velocity-dependent damping factor. However, a reduction in muscle activity of the gluteus maximus can be observed again, indicating that the applied forces are assisting with the hip extensors.

### 4.3. Potential users of the myosuit

The Myosuit was conceived to assist a large population of people with various degrees of muscle weakness. This includes musculoskeletal and neurological disorders, for example, incomplete spinal cord injury (SCI). SCI has an incidence of about 15–40 cases per million (Sekhon and Fehlings, 2001). Depending on ASIA grade (A–D) (Sekhon and Fehlings, 2001), SCI patients are able to stand up and walk. ASIA Grades C and D include patients with incomplete SCI that maintain full range of motion and the ability to move against gravity with at least half of the key muscles. These abilities, however, are limited for most of the patients (Barbeau et al., 1999). A survey with paraplegic patients found that mobility concerns are more prevalent than other life areas (Heinemann et al., 1987). The Myosuit will be most beneficial in patient groups that have problems with upright stability and movements that rely strongly on the anti-gravity muscles.

The Myosuit was not designed to provide the forces that correspond to 100% of the biological joint torques. The system instead requires the user to have remaining muscle functionality and work with the system to perform the desired movements. Although, it has been demonstrated that the posture estimation using the Myosuit's stiffness worked in one subject, it has to be proven in many more subjects to validate the widespread efficacy of the assistance.

### 4.4. Outlook

This article introduces the design of the Myosuit and its layered, textile interface. In addition, a virtual leg based anti-gravity control concept was evaluated on one subject for sitting transitions. Further experiments with an increased number of subjects are required to further evaluate the promising potential of the Myosuit and reach a conclusion on its ability to reduce the muscle activity of its users. The experiments should be conducted with improved force scaling to apply forces up to 435 N, and more advanced triggers as well as advanced damping approaches should be implemented.

The Myosuit is a lightweight untethered exosuit for bi-articular assistance of hip and knee extension, that includes a novel antagonistic design (power vs. ligament layer) to overcome the limitation of existing exosuits that only apply assistance in one direction per joint. The use of the ligament layer was not needed during sitting transfer and therefore not used in this study. This specific design feature needs further investigation.

To extend the concept to walking it is necessary to disable the anti-gravity control during swing phase. First tests used the shank angle and a fixed timing based on a knee extensor moment from literature (Grimmer and Seyfarth, 2014), to enable and disable the anti-gravity control during gait. It is planned to further generalize this concept by using a phase plane controller of the shank (Holgate et al., 2009) or a virtual leg (Villarreal and Gregg, 2014). The virtual leg length could be used for scaling assistance forces and the virtual leg kinematics to enable assistance in stance and disable assistance in flight. During swing, the ligament layer could then compensate for some of the weight of the leg.

## Author contributions

The concept of the Myosuit was developed by KS and RR; The electronics and actuators were designed by KS; The high level control was designed by KS, MG, AS, and HW; Low level controls were design and implemented by KS; The study was designed by MG, JD, and KS, MG, and JD performed the experiments; Data analysis and interpretation was performed by KS, MG, CE, and JD; KS, JD, and MG were responsible for drafting the article. All authors revised the article. All authors gave final approval of the version to be submitted and any revised version.

### Conflict of interest statement

The authors declare that the research was conducted in the absence of any commercial or financial relationships that could be construed as a potential conflict of interest.
